# HMPLMD: Handwritten Malayalam palm leaf manuscript dataset

**DOI:** 10.1016/j.dib.2023.108960

**Published:** 2023-02-08

**Authors:** B.J. Bipin Nair, N. Shobha Rani

**Affiliations:** Department of Computer Science, Amrita School of Computing, Mysuru, Amrita Vishwa Vidyapeetham, India

**Keywords:** Binarization, Malayalam, Sauvola, Ground truth, Photoshop

## Abstract

The realization of high recognition rates of degraded documents such as palm leaf manuscripts primarily relies on document enhancement. Advancement of deep learning models in the process of document enhancement plays a major role among non-deep learning models or thresholding methods. Preparation of readily available ground truth data for creation of deep learning models is of paramount importance as it is highly time consuming task. The ground truth dataset preparation involves greater complexities as ancient documents are affected with degradations such as fungi, humidity, uneven illumination, discoloration, holes, cracks, and other damages. We propose a Handwritten Malayalam Palm Leaf Manuscript Dataset (HMPLMD) and its ground truth data aspiring for advancements in the field of palm leaf image analysis. We employ the palm leaf manuscripts of Kambaramayanam and Jathakas for the sake of experimentations. The proposed ground truth samples of degraded palm leaves plays a crucial role in creation of specialized deep/transfer learning models to handle challenges related to binarization.

**Specifications Table**

**Table:** 

Subject	HMPLMD: Handwritten Malayalam Palm Leaf Manuscript Dataset
Specific subject area	Palm leaf manuscript enhancement, document binarization;
Type of data	Table Image Figure
How the data were acquired	A group of four individuals have been deployed to capture the datasets. Entire dataset is splitting into two groups to adapt two different imaging modalities during image acquisition. The first group of palm leaf datasets are acquired using NIKON D3400 DSLR camera mounted on a tripod and iPhone 13 mounted on a tripod for the second group of datasets. The manuscripts are arranged in an ordered sequence in batches of two to four manuscripts to take the pictures from each bundle. After all the images are captures, the digitized images are segregated into two folders based on imaging modality followed. Later images are split into individual palm leaves using cropping tool of image editing software.
Data format	Raw palm leaf manuscripts with degradations and its cleaned samples in .jpg format.
Description of data collection	Palm leaf samples are belonging to collections of Kambaramayanam (151) and Jathakas (99) from ancient temples and astrologers from Palakkad, Kerala. The samples are highly affected by multiple degradations that are naturally formed due to biological activity, climatic conditions and document aging. Samples are carefully chosen to encompass all the degradation types and multiple issues related to aging of palm leaf documents.
Data source location	Kambaramayanam- Kerala- Palakkad- Nedumpalli Mana. Jathakas- Kerala- Kannur- Nandith House.
Data accessibility	doi:10.17632/ssycxg2snx.1
Related research article	Shobha Rani, N., Bipin Nair, B. J., Chandrajith, M., Hemantha Kumar, G., & Fortuny, J. (2022). Restoration of deteriorated text sections in ancient document images using a tri-level semi-adaptive thresholding technique. *Automatika*, *63*(2), 378-398. https://doi.org/10.1080/00051144.2022.2042462

## Value of the Data

**Fig. 1:**
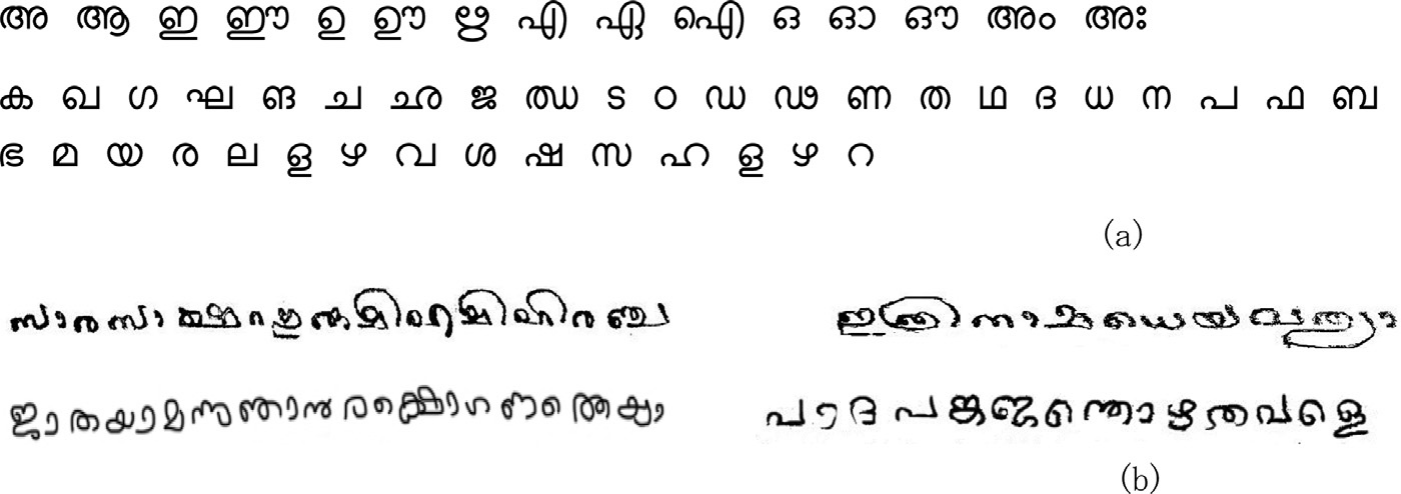
(a) Malayalam vowels and consonants (b) Word level samples.
B **Knowledge transfer**
  • Jathakas – The datasets consist of diagrams representing various planetary alignments at the time of birth of a person. These Manuscripts often describe the position of the Sun, Moon, Planets, other Astrological aspects, and sensitive angles at the time of the birth. Based on this document, a person knowing Astrology can predict the future of the person about whom the Jathaka was written. The manuscripts deal with not only a person's life cycle; it includes a place of origin, a temple history, and many mathematical calculations.
  • Ramayana – It was initially composed in Sanskrit and was later translated into most of the Indian languages. The first writings of the Ramayana were estimated to be written between the 4th and 7th BCE and are one of the essential epics of Hinduism.
  • Kambaramayanam – Samples consist of the essence of the Valmiki's Ramayana and was written during the 12th century by a poet named Kambar. It was originally written in Tamil and was later on rewritten in Malayalam. In kambaramayanam depicts the actual Ramayana story, but the new versions, which are exciting now, are the edited and modified versions.
  • Deploying the Transfer learning approach upon the binarized documents helps generate extensive data from the older Malayalam characters. Each document was collected from different parts of Kerala, leading to a good number of variations in the dataset, which can help generate an efficient and robust learning model for degraded document binarization. Further, the datasets aid in converting unknown palm leaf manuscripts into their binarized form leading to excellent readability and accessibility that have substantial societal impact. Using these ground truth images, we can denoise other language manuscripts via transfer learning approach.

A **Technical contribution**
  • This data is beneficial for developing palm leaf manuscript binarization algorithms using deep/transfer and machine learning algorithms.
  • The datasets are also employable for development of semantic segmentation algorithms for palm leaf manuscripts. Additionally, ground truth samples can be employed for creation of character or word datasets for Malayalam script that are directly employable for character recognition tasks.
  • The datasets help create deep learning pre-trained models for characterizing noise type variants in ancient documents.
  • The ground truth data set are useful in extraction of character datasets composed of vowels, consonants, and compound characters useful in handwritten Malayalam character recognition as well as word level samples. (see Fig: 1).

## 1 Objective

**Fig. 2:**
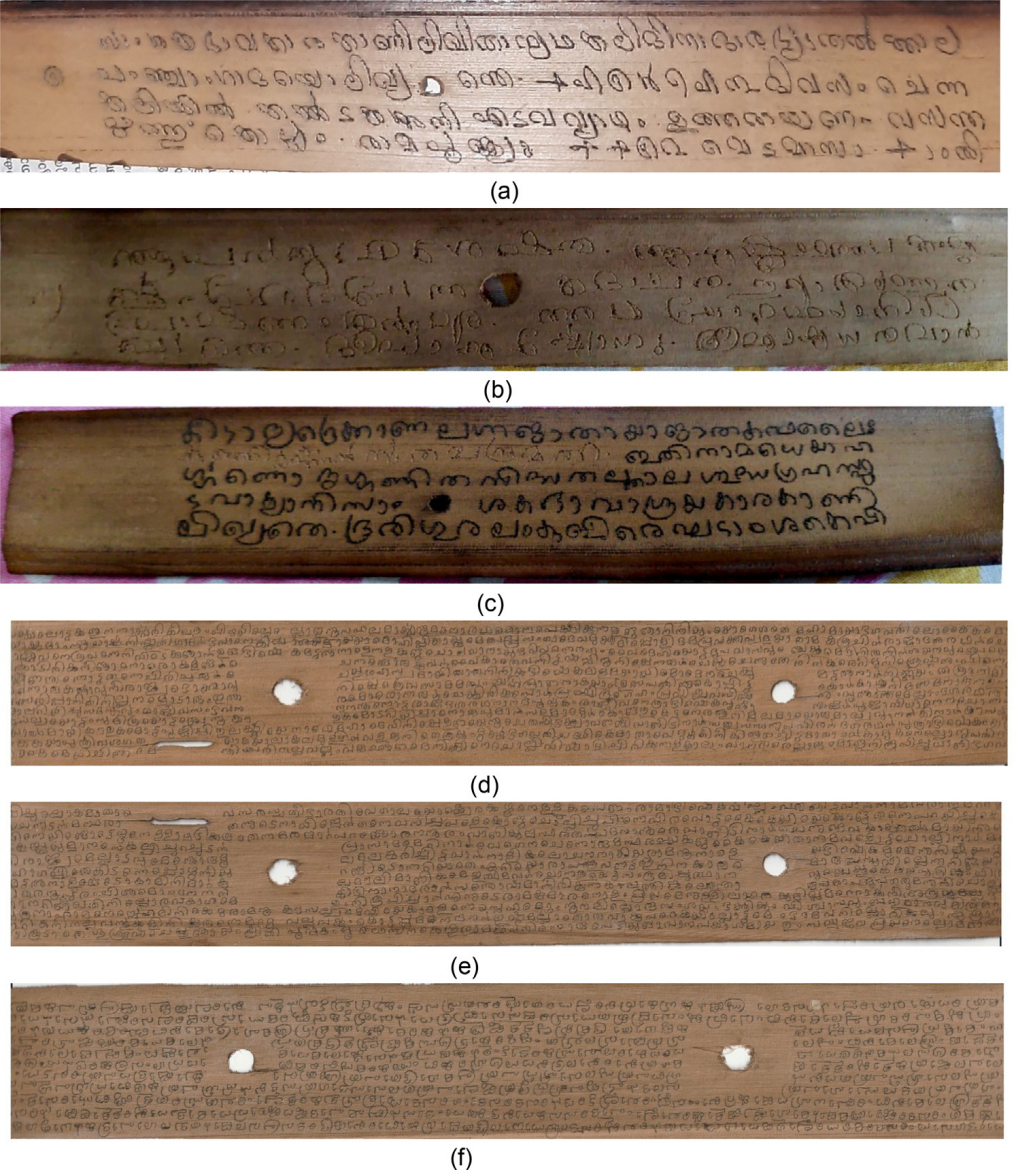
(a) Sample 1- Jathakas (b) Sample 2- Jathakas (c) Sample 3- Jathakas (d) Sample 1-Kambaramayanam (e) Sample 2- Kambaramayanam (f) Sample 3- Kambaramayanam.

India has a rich social and cultural past that spans centuries. Ancient knowledge is reposited with text inscription on dried and processed palm leaves. Ancient documents and manuscripts containing cultural, historical, and medical information cover our rich and old culture in great detail. Most of these manuscripts are written on palm leaves, prone to deterioration from improper handling, moisture exposure, and fungus development. The palm leaf manuscripts comprise many records, from common texts to religious compositions. Epics like the Mahabharata, Ramayana, Kambaramayanam and even astrological scripts like Jathakas are available on palm leaves. Most of these documents are in museums, private collections and other public libraries which are in degraded form due to poor maintenance conditions [1]. The texts on these manuscripts were created mainly with a stylus-like tool called "Ezhuthani," dipped in dyes and used to color the scratched leaves. Text inscribed on palm leaf manuscripts are written with variable character or word or line spacing, making them difficult to read [2]. The dataset's deficiencies like holes, cracks, termites attacking problem, discoloration, ink bleed through and uneven illumination complicate the digitization process [3] (see Fig. 2).

The primary objective of this work is to contribute datasets of palm leaf manuscripts related to Kambaramayanam and Jathakas along with the ground truth data. The proposed contribution is to promote the development of a deep/transfer/machine learning algorithms for document binarization for ancient document repair and facilitate data extraction for information processing. As development of pre-trained models using deep/transfer /machine learning models relies greatly on the learning resources of the specific problem domain. With regard to palm leaf manuscript analysis, the removals of degradations that occur due to climatic, biological and aging factors are trivial. Resolution of degradation issues concerning palm leaf manuscripts using thresholding or parameter dependent methods are much sensitive to exceptions. As a result, automated solutions proposed based on the learning resources related to degradations of palm leaf manuscripts introduces the reliability of the document binarization algorithms.

The proposed datasets along with the ground truth data named HMPLMD dataset encompassing multiple degradation issues that are commonly found in palm leaf manuscripts. The ground truth images corresponding to its original images are instrumental in training of deep/transfer/machine learning models. The deployment of the proposed datasets as part of learning repositories for afore-mentioned models aggravates quality in terms of manuscript digital quality by reducing algorithms sensitivity towards errors. The proposed HMPLMD dataset and few samples of palm leaves are presented in Table 1.

**Table 1:** Dataset collections: category and source.

Description	Document characteristics	Number of Original images	Number of Ground truth images	Source
Kambaramayanam- Ancient Indian epic	Discoloration, cracks, blurring, fungus, humid condition	151	24	doi:10.17632/ssycxg2snx.1
Jatakas-Birth Charts & Horoscope	Discoloration, cracks, blurring, fungus, uneven illumination	99	45	doi:10.17632/ssycxg2snx.1

## 2 Data Description

### 2.1 Technical knowledge contribution to ancient document image analysis

**Fig. 3:**
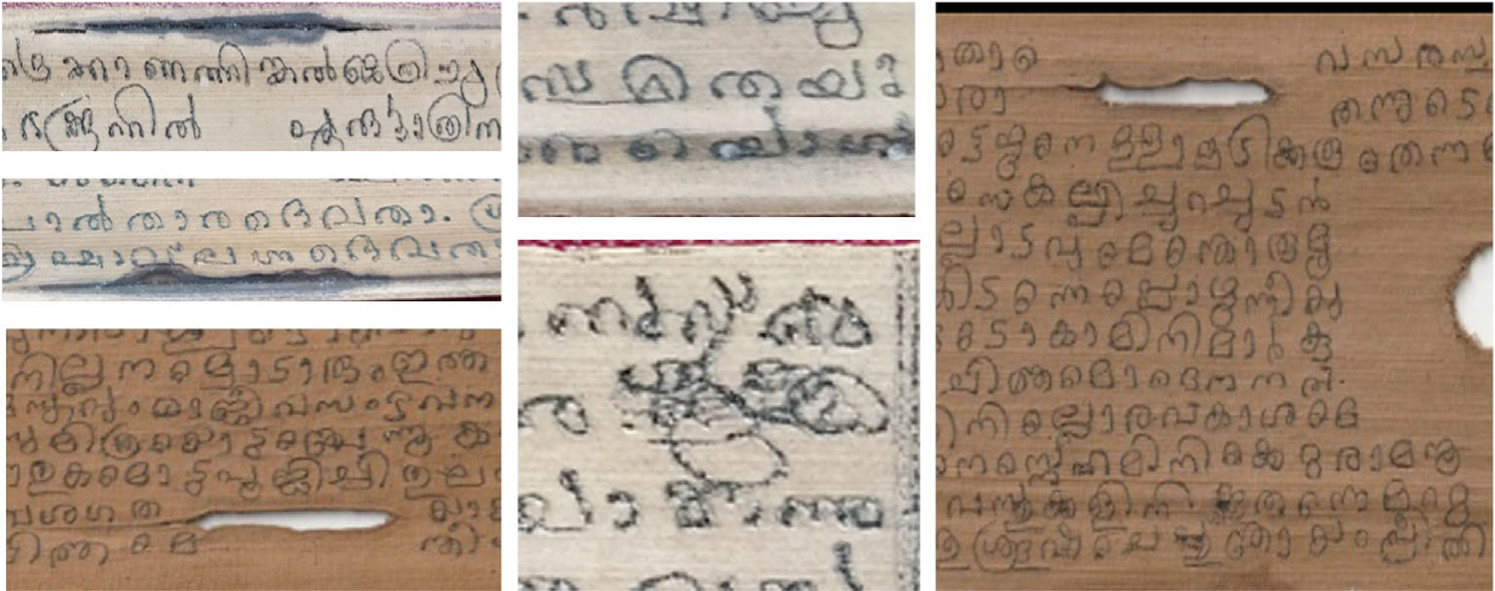
Degradation's from the documents.

In the existing repositories related to palm leaf manuscripts, most of the datasets of Malayalam are from a single source named Shiju Alex. The datasets from Shiju Alex are primarily related to Pazhayannur sthuthi, Tantra shastra, and Krishnapattu. These datasets are available in raw form and are not being supplemented with any additional ground truth data. Furthermore, without ground truth datasets, these datasets are not deployed for implementation of deep/transfer/machine learning models. Therefore, supplementing the palm leaf manuscripts with the metadata useful for developing OCR-related protocol leading to significant advancements in the field of ancient document analysis. Therefore, this work aims to create ground truth metadata of the degraded Malayalam palm leaf manuscripts. Some of the samples related to Jathakas and Kambaramayanam are presented in Fig 2 and some of the degraded samples of Jathakas and Kambaramayanam are presented in Fig. 3.

Table 1 describes the dataset collection along with the description, characteristics and other statistics in terms of number of samples and its accessibility. The dataset collected include two categories: ancient Kambaramayanam, which represents the mythological epic of Ramayana with 151 images acquired from Nedumpalli Mana Palakkad, Kerala. The other category, Jathakas describes the astrological details about various people's life, temple history, place with 99 samples from Kannur, Kerala. Altogether, 250 raw images with 69 ground truth samples are available via link: doi:10.17632/ssycxg2snx.12.

## 3 Experimental Design, Materials and Methods

### 3.1 Data collection

The authors collected the datasets from various places in Kerala, India as described in Table 1. Online repository of all raw samples is accessible via doi:10.17632/ssycxg2snx.1.

### 3.2 Data acquisition

**Fig. 4:**
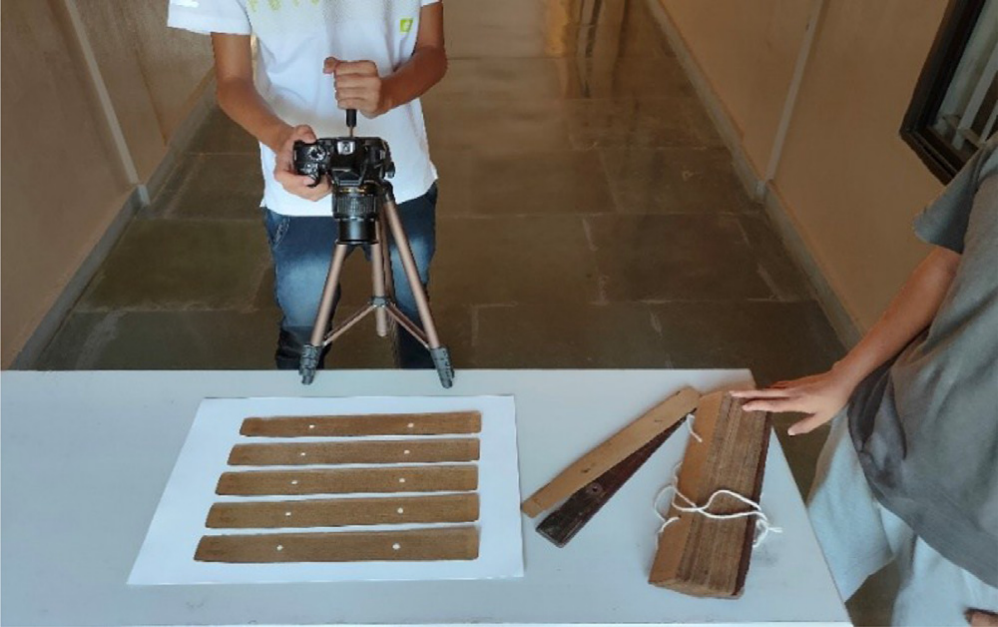
Dataset capturing setup.

All the data procured were captured by splitting into two batches. While one batch is acquired using a Nikon D3400 DSLR camera, the other uses an iPhone 13 camera mounted on a tripod (see Fig. 4). Proper lighting was provided for better visual quality of the palm leaf manuscript. Both groups of images are acquired by considering five leaves as one batch arranged in horizontal sequential order, as shown in fig. 4. All the images are captured by providing sufficient lighting required for text legibility. Using the below arrangement, we captured around 250 datasets of Kambaramayam and Jathakas.

### 3.3 Data pre-processing

All the scanned documents were visually inspected to ensure no unclear or blurred images. The scanned images were then divided into groups for pre-processing. The images were run through Photoshop for curve-fitting adjustment and white balancing, which were then binarized using Sauvola thresholding. Using the brush tool, the individual then ran the binarized images through Photoshop to manually write/verify the missing letters. The final ground truth image was saved in JPEG/PNG format. Further, each image was subsampled into five separate images and stored.

**Fig. 5:**
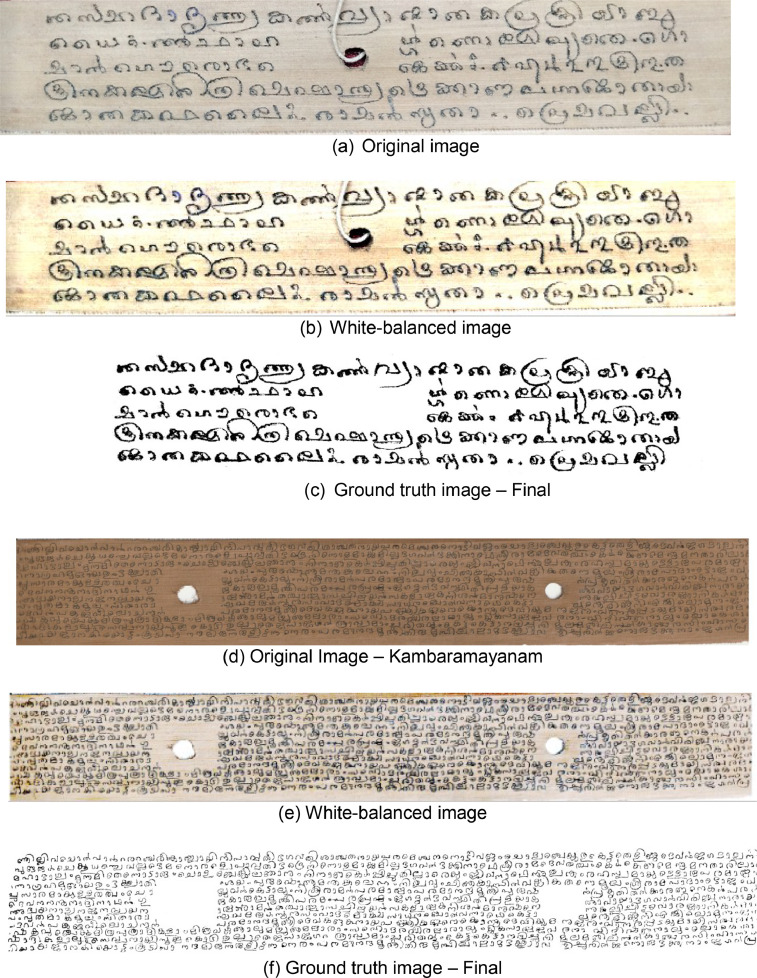
Samples of ground truth images (a) Original image (b) White-balanced image (c) Ground truth image – Final (d) Original Image – Kambaramayanam (e) White-balanced image (f) Ground truth image – Final.

The above Fig. 5 (a) to (f) shows the experimental results in the order of the original image, the White-balanced image, and the ground truth image-Final of the two categories of datasets Kambaramayanam and Jathakas. To obtain ground truth images, initially, samples of palm leaf images captured are subject to basic enhancement operations using imaging editor such as Adobe Photoshop and then using adaptive binarization methods such as, Sauvola [4], Niblack [5] and Wolf [6] as per the degradation to be removed. In post enhancement stage, we use a brush and eraser tool in adobe Photoshop to remove some partial degradation patterns that are not removed using adaptive thresholding methods to get the final ground truth image.

## Ethics statements

This work does not involve studies with animals and humans.

## CRediT authorship contribution statement

**B.J. Bipin Nair:** Conceptualization, Formal analysis, Investigation, Data curation, Methodology, Resources, Writing – review & editing, Writing – original draft, Validation. **N. Shobha Rani:** Conceptualization, Formal analysis, Investigation, Data curation, Methodology, Resources, Writing – review & editing, Writing – original draft, Validation, Supervision.

## Declaration of Competing Interest

The authors declare that they have no known competing financial interests or personal relationships which have or could be perceived to have influenced the work reported in this article.

## Data Availability

Ancient palm leaf documents (Original data) (Mendeley Data).

## References

[1] Fischer Andreas, Indermühle Emanuel, Bunke Horst, Viehhauser Gabriel, Stolz Michael (2010). *Proceedings of the 9th IAPR International Workshop on Document Analysis Systems*(*DAS '10*).

[2] Kesiman M.W.A., Burie J.-C., Wibawantara G.N.M.A., Sunarya I.M.G., Ogier J.-M. (2016). 2016 15th International Conference on Frontiers in Handwriting Recognition (ICFHR).

[3] Shobha Rani N., Sajan Jain A., Kiran H.R., Pandian D., Fernando X., Baig Z., Shi F. (2019). Proceedings of the International Conference on ISMAC in Computational Vision and Bio-Engineering 2018 (ISMAC-CVB).

[4] Sauvola J, Pietikainen M. (2000). Adaptive document image binarization. Pattern Recognit..

[5] Niblack W (1986).

[6] Christian Wolf, Jolion JM, Chassaing F (2002). Object Recognition Supported by User Interaction for Service Robots.

